# Evaluating Challenges and Adoption Factors for Active Assisted Living Smart Environments

**DOI:** 10.3389/fdgth.2022.891634

**Published:** 2022-05-30

**Authors:** Lena Lam, Laura Fadrique, Gaya Bin Noon, Aakanksha Shah, Plinio Pelegrini Morita

**Affiliations:** ^1^School of Health, University of Waterloo, Waterloo, ON, Canada; ^2^Research Institute for Aging, University of Waterloo, Waterloo, ON, Canada; ^3^Department of Systems Design Engineering, University of Waterloo, Waterloo, ON, Canada; ^4^eHealth Innovation, Techna Institute, University Health Network, Toronto, ON, Canada; ^5^Institute of Health Policy, Management, and Evaluation, Dalla Lana School of Public Health, University of Toronto, Toronto, ON, Canada

**Keywords:** Active Assisted Living (AAL), aging in place, smart city, ambient assisted living, smart living

## Abstract

While there have been rapid advancements in individual technologies such as Internet of Things (IoT) and Active Assisted Living (AAL) to address challenges related to an aging population, there remain large gaps in how these technologies can be integrated into the broader ecosystem to support older adults in aging in place. This research provides an overview of 15 solutions available to date around the globe and compares key factors for adoption in each solution, including user acceptance, privacy and security, accessibility, and interoperability. To scale these solutions sustainably and universally, the development and implementation of standards for key factors for adoption in AAL environments is critical. There is also a need for increased and sustainable funding to complement research priorities, to continue advancing AAL technologies.

## Introduction

Global population aging is increasing dramatically at an unprecedented pace. It is estimated that the proportion of the world's population over 60 will nearly double from 12 to 22%, between 2015 and 2050 (1). This demographic shift presents challenges to all countries, such as ensuring healthcare and social support systems are adept to meet the increasing needs and demands of an aging population.

The current literature indicates that well-designed technological solutions may act as facilitators to help address challenges related to an aging population (2). As the development of technologies such as Internet of Things (IoT) and Active Assisted Living (AAL) technologies continue to advance, it is anticipated that these innovations will influence future architecture and infrastructure development, giving rise to more smart communities and cities, offering both sensing and actuating capabilities such as assistive interventions for older adults (3–5). Advances in technology can aid in preventing, educating, and monitoring behaviors to assist older adults with their activities of daily living (ADL) and support them in learning and remembering healthy behaviors (6). Furthermore, technologies can help older adults remain in the community, shifting to a more decentralized model of care, which can present cost-savings for healthcare systems, yet more effective patient-centered care (7).

While there have been promising advancements, there is limited research available on the key challenges in scaling assistive communities to meet the needs of older adults for active and successful aging. In this article, we provide an overview of the key factors for adoption of AAL technologies and highlight 15 case studies of solutions from around the globe. As a secondary insight, we provide a comparison between the state of AAL globally compared to within the Canadian context.

## Materials and Methods

In this research, a narrative literature review was conducted followed by a document analysis to synthesize the current state of knowledge of AAL systems. For the narrative review, select papers included those published in peer-reviewed journals in English. The data sources included Google Scholar, PubMed, PsycINFO, Cochrane Library, and Scopus. The inclusion criteria for the research focused on AAL communities in any stage of deployment that assessed or mentioned at least one of the following factors: user acceptance, privacy and security concerns, accessibility, and interoperability, which are factors derived from Design for All framework (8, 9). The Design for All framework is a holistic paradigm that considers a broad spectrum of human diversity (9). It proposes flexible designs, offering universal features easily adapted to the needs of a specific user (9).

For the document analysis, documents from Google, including reports published by institutions, industry, governments, and independent research groups, were analyzed. Books, background papers, program proposals, application forms, summaries, and manuals were also included in the analysis.

The four factors selected for comparison and analysis of the AAL communities have been previously identified in the literature as key factors for consideration when deploying AAL systems. The themes for analysis are described below.

### User Acceptance

User acceptance is an important aspect for the successful adoption of health technologies. Acceptance of new technologies depends on perceived risks, including whether the technology delivers services in a secure, reliable, and effective manner (10, 11). The results in Offermann-van Heek et al. suggested that “personal care needs” were a parameter that potentially influenced AAL technology acceptance in older adults (12, 13). Higher needs for care would lead to higher acknowledgments of the technology's benefits.

### Privacy and Security

Privacy and security issues have been widely cited as concerns by users as AAL technologies monitor, communicate and provide services based on a full-time surveillance basis (14). AAL systems collect and analyze an immense amount of sensitive information about users, including medical and behavior pattern information (15). The use of privacy protection standards is important for respecting a person's autonomy, as well as for building and promoting trust (16). Standards and methodologies such as Privacy by Design should be utilized during the early stages of a system design. This helps ensure that end user privacy concerns are taken into consideration and influence the design of the overall system architecture (17).

### Accessibility

Accessibility refers to the degree to which different entities such as a device, interface, resource, system, environment can be used and provide a benefit for as many people as possible (18). The term “accessibility” is often used in the context of people with physical disabilities and their right to access available products and services. However, accessibility is influenced by a multitude of interconnected factors ranging many levels of impact, such as physical, mental, behavioral, social, and environmental (18). It is imperative that AAL systems have a broad range of intelligent functions to support usable and accessible interfaces with adaptive mechanisms for user interactions (19).

### Interoperability

The current landscape of AAL systems and environments is highly fragmented due to a lack of reference standards in AAL systems and competition between different vendors (20). Interoperability is recognized as a key requirement for the deployment of successful products in AAL environments as systems and devices must be highly integrated to provide users with comprehensive and effective services (21). In addition, there are upfront costs associated with implementing AAL systems, including but not limited to hardware and software costs and installation fees. From a user's perspective, it is important that the AAL systems are “future-proof” in terms of the possibility to grow and adapt to users' changing needs over the years (21, 22).

## Results

### Smart Living Environments (SLEs) Around the Globe

The results include an analysis of 15 smart living environments around the globe; six located in Europe, six located in North America and the remaining three located in Asia (Table 1). Of the 15 environments, five are smart homes, five are smart communities and five are smart cities (Table 2). Six of the environments were pilots, which are now inactive; five are currently in development; three are currently operational and active and one project was canceled (Table 1).

**Table 1:** Summary tables of SLEs.

**Categories**	**Sub-categories**	Total^*^/(%)^**^
Type of smart living environments	Smart home	5/33%
	Smart community	5/33%
	Smart city	5/33%
Status of smart living environments	Pilots (inactive)	6/40%
	In development	5/33%
	Operational (active)	3/20%
	Canceled	1/7%
Geographical location of smart living environments	Europe	6/40%
	North America	6/40%
	Asia	3/20%
Funding and ownership of smart living environments	Publicly owned or funded (i.e., research institutions, universities or governments)	5/33%
	Privately owned and solely funded by technology corporations	3/20%
	Owned and funded by public institutions with partnerships with private partners	7/47%
Priority for health and AAL in smart living environments	Health as a priority	13/86%
	AAL as a priority	7/46%

* *Number of smart living environments that fit the category/sub-category*.

** *Percentage based on 15 total smart living environments reviewed*.

**Table 2:** Overview of 15 SLEs.

**No**.	**Type**	**Smart living environment**	**Continent**	**Level of readiness**	**Type of project owner**	**Consideration for health**	**Consideration for AAL**
1	Home	Make it REAAL	Europe	Pilot (inactive)	Public	Yes	Yes
2	Home	Vesta	Europe	Pilot (inactive)	Public	Yes	No
3	Home	SOPRANO project	Europe	Pilot (inactive)	Public	Yes	Yes
4	Home	HomeSense	North America	Pilot (inactive)	Public	Yes	Yes
5	Home	UbiCare	Europe	Pilot (inactive)	Public	Yes	Yes
6	Community	Fujisawa sustainable smart town	Asia	Ready (active)	Private	Yes	Yes
7	Community	Montreal	North America	In development	Public with private partners	Yes	No
8	Community	Edmonton	North America	In development	Public with private partners	Yes	N/A
9	Community	The orbit	North America	In development	Public with private partners	Yes	N/A
10	Community	Drayton valley	North America	Pilot (inactive)	Public with private partners	No	No
11	City	Woven city	Asia	In development	Private	Yes	Yes
12	City	Sidewalk labs Toronto Quayside	North America	Canceled	Private	No	No
13	City	U-City	Asia	In development	Public with private partners	Yes	Yes
15	City	Barcelona	Europe	Ready (active)	Public with private partners	Yes	No
15	City	Amsterdam	Europe	Ready (active)	Public with private partners	Yes	No

### Project Ownership

Within the 15 smart living environments, five are publicly owned and funded either by research institutions, universities, or governments. Three are privately-owned and solely funded by technology corporations. Lastly, seven are owned and funded by public institutions with partnerships with private partners to provide technological capacity and infrastructure (Table 1).

### Priority for Health and AAL

Out of the 15 smart living environments, 13 had health as a priority. Of those thirteen, five are smart homes, four are smart communities and four are smart cities (Table 1). Beyond health, AAL was a priority in four smart homes, one smart community and two smart cities (Table 1). Technologies included in smart living environments enabling health and AAL include both ambient and wearable sensors to help visualize daily activity and provide real time notification systems to signal adverse events (22, 23).

### SLE Analysis for Factors of Adoption

#### User Acceptance

The level of publicly available information and understanding of user acceptance varied between project owners and funders. Most notably, there was a lack of publicly available information on user acceptance for privately-owned projects that are solely funded by technology corporations. This could potentially be due to non-disclosure agreements between residents and the technology companies.

Regarding end-user testing to understand user acceptance, the richness of data decreased as project sizes increased. At the smart home level, all projects conducted some form of end user consultation and end users were involved in testing in a real-life setting. End users for all five smart home projects were also involved in the early scoping phases. In three out of five projects, user input was heavily considered throughout the entire development process of the platform to iterate on service offerings, prior to implementation (Table 3). Two smart home projects used a framework to evaluate user needs, including the Technology Acceptance Model and The Smart Home Technology Acceptance Model (Table 3).

**Table 3:** User acceptance results.

**User acceptance factors**	N^*^/(% of total)^**^	**Smart living environments**
Conducted end user testing	5/33%	Make it REAAL, Vesta, SOPRANO Project, HomeSense, UbiCare
User input heavily considered throughout development	3/20%	Make it REAAL, SOPRANO and HomeSense
Formal user acceptance framework utilized	2/13%	The Make it REAAL project SOPRANO Project
Public consultation in the beginning scoping phases	3/20%	Montreal in Quebec, The Orbit in Innisfil and Drayton Valley in Alberta
Platform to collect ongoing user feedback	1/7%	Barcelona

* *Number of smart living environments that fit the category/sub-category*.

** *Percentage based on 15 total smart living environments reviewed*.

*Total N > 100% as smart living environments could have had multiple factors related to user acceptance*.

As the project sizes increased, smart community and city projects had less extensive end user feedback collection cycles, as well as less end user testing protocols. For example, public consultations only occurred in the beginning scoping phases to understand needs and project focus. Three communities collected public responses in the early development phase (Table 3). Only one project developed a platform to collect ongoing feedback from citizens to iterate on service offerings and understand user needs (Table 3). In most cases, it is unclear how public feedback is incorporated into the development and iteration of services as smart living environment projects get larger.

#### Privacy and Security

As smart living environments collect, retain, and analyze large volumes of personal data, privacy and security become major consideration. Ownership of data, technologies used to ensure encryption and regulations, or policies in place are key themes that emerged when analyzing privacy and security.

##### Ownership of Data

The data custodian refers to an entity that oversees the storage, aggregation, and use of data sets. In this research, it was found that the primary data custodian oftentimes is dependent on the project owner and the main funder. The results showed that 12 environments were governed by public institutions while 3 environments were governed by a private system. In the smart home projects, data was primarily collected, analyzed, and governed by research teams or public institutions, such as governments (Table 4). In three smart community projects, there were dedicated governing bodies responsible for data ownership.

**Table 4:** Privacy and security results—type of data custodian and project lead.

**Type of data custodian and project lead**	N^*^/(% of total)^**^	**Smart living environments**
Governed by public institution	12/80%	Make it REAAL, Vesta, SOPRANO Project, HomeSense, UbiCare, Montreal, Edmonton, The Orbit, Drayton Valley, U-City, Barcelona, Amsterdam
Governed by private and proprietary system	3/20%	Fujisawa Smart Town, Woven City, Sidewalk Labs Toronto Quayside

* *Number of smart living environments that fit the category/sub-category*.

** *Percentage based on 15 total smart living environments reviewed*.

##### Technological Elements to Security

There were also different technological methods deployed to encrypt data and keep data secure and private. At the smart home level, technological elements were used in the system's security setup, such as Zigbee and OSGI. Zigbee addresses basic privacy requirements in AAL systems and is a specification that supports message confidentiality, integrity on network and application layers (23). OSGI provides a general purpose, secure support for deploying Java-based service applications, which is utilized in smart living environments (24). In one project, called the Ubicare project in Europe, developers did not use cameras or microphones, as these devices are commonly perceived as privacy violators (23). Rather, unobtrusive sensors were integrated into the physical environment to be minimally invasive (23).

##### Policies and Regulation

In terms of regulations and policies, there were many methods utilized across the smart living environments. For example, two environments conducted Privacy Assessment Impacts (PIAs) while developing smart environment plans. Only one smart home was Health Insurance Portability and Accountability Act (HIPAA) compliant among the environments collecting health information. Some environments adhered to various protocols including the Privacy by Design approach, the Freedom of Information and Protection of Privacy (FOIP) Act, and the General Data Protection Regulation (GDPR) (Table 5).

**Table 5:** Privacy and security results—polices and regulations related to privacy and security.

**Regulation/protocol**	N^*^(% of total)^**^	**Smart living environments**
Privacy impact assessments	2/13%	Make it REAAL, Edmonton
Health insurance portability and accountability act	1/7%	HomeSense
Privacy by design	1/7%	Edmonton
Freedom of Information and Protection of Privacy (FOIP) ACT	2/13%	Edmonton, Drayton Valley
General data protection regulation	2/13%	Amsterdam, Barcelona
N/A	9/60%	Vesta, SOPRANO Project, UbiCare, Fujisawa Sustainable Smart Town, Montreal, The Orbit, Woven City, U-City

* *Number of smart living environments that fit the category/sub-category*.

** *Percentage based on 15 total smart living environments reviewed*.

*Total N > 100% as smart living environments could have had multiple polices and regulations related to privacy and security*.

##### User Autonomy

Ten environments considered the security, and autonomy of users. When considering end user autonomy and respecting privacy, only three environments explicitly offered users the ability to opt-out of data sharing and collection (Table 6). Out of the 15 environments, only one did not consider privacy and security implications before implementation while four did not have any information available on their privacy and security policies (Table 6).

**Table 6:** Privacy and security results—consideration for user autonomy and privacy.

**Privacy, security and autonomy of users**	N^*^/(% of total)^**^	**Smart living environments**
>1 Consideration for privacy, security and autonomy of users	10/67%	Make it REAAL, SOPRANO Project, HomeSense, UbiCare, Fujisawa Sustainable Smart Town, Edmonton, Drayton Valley, Sidewalk Labs Toronto Quayside, Barcelona, Amsterdam
Offered ability to opt-out of data sharing and collection	3/20%	HomeSense, Fujisawa, and Sidewalk Labs
No consideration of privacy, security and autonomy of users	1/7%	U-City
N/A	4/26%	Vesta, Montreal, The Orbit, Woven City

* *Number of smart living environments that fit the category/sub-category*.

** *Percentage based on 15 total smart living environments reviewed*.

*Total N > 100% as smart living environments could have had considerations for privacy, security and autonomy as well as offered ability to opt-out of data sharing*.

#### Accessibility

There was limited data in the literature discussing accessibility as a priority in smart living environment strategies. In total, six environments had a strategic priority for accessibility (Table 7). Two smart city initiatives—Edmonton (Canada) and Quayside Sidewalk Labs (Canada) had a strategy for inclusive engagement. Moreover, Edmonton had a priority to create a smart city that considered the needs of marginalized groups such as newcomers to Canada, urban Indigenous population, seniors, children, and youth, as well as people living in poverty and homelessness (25). Sidewalk Labs had a priority that combined inclusivity, accessibility, and equitable measures for digital literacy efforts to promote the skills to use the proposed resources (26) (Table 7). Only two smart home projects, evaluated service and interface usability while four smart home projects were more cost-conscious and energy-efficient for end users (Table 7).

**Table 7:** Accessibility results.

**Consideration for accessibility**	N^*^/(% of total)^**^	**Smart living environments**
Evaluated service or interface usability	2/13%	Vesta, SOPRANO
Cost conscious development	4/27%	Make it REAAL, Vesta, SOPRANO Project, Ubicare
Strategy for inclusive engagement	6/40%	Make it REAAL, Vesta, SOPRANO Project, HomeSense, Edmonton, Quayside Sidewalk Labs
N/A	8/53%	Fujisawa Sustainable Smart Town, Montreal, The Orbit, Drayton Valley, Woven City, U-City, Barcelona, Amsterdam

* *Number of smart living environments that fit the category/sub-category*.

** *Percentage based on 15 total smart living environments reviewed*.

*Total N > 100% as smart living environments could have had multiple factors and consideration points related to accessibility*.

#### Interoperability

Five smart living environments utilized technological elements, such as a middleware, to create an interoperable network for sensors to provide the end user with a comprehensive service (Table 8). Make it REAAL was focused on building their own open-source middleware to provide interoperable services to end users (27). HomeSense utilized an array of networked wireless Z-Wave devices and a Raspberry Pi connected to the internet as a remote gateway (28). Vesta used four Raspberry Pis as gateways and one 4G router (29). Ubicare used Arduino microcontrollers coupled with ZigBee-compatible XBee RF networking modules for wireless communication among the nodes (23). SOPRANO used an open platform based on a combination of semantic-enabled technologies and service-orientation (30). Furthermore, four environments had interoperability as a strategic priority outlined in their strategies and plans (Table 8).

**Table 8:** Interoperability results.

**Consideration for interoperability**	N^*^/(% of total)^**^	**Smart living environments**
Utilize technological elements in system setup related to interoperability (i.e., middleware)	5/33%	Make it REAAL, Vesta, SOPRANO Project, HomeSense
Strategic priority	4/27%	Make it REAAL, Montreal, Sidewalk Labs, U-City
N/A	7/47%	Fujisawa Sustainable Smart Town, Edmonton, The Orbit, Drayton Valley, Woven City, Barcelona, Amsterdam

* *Number of smart living environments that fit the category/sub-category*.

** *Percentage based on 15 total smart living environments reviewed*.

*Total N > 100% as smart living environments could have utilized technological elements and had a strategic priority for interoperability*.

#### Strategic Priority for Health and AAL

As project sizes increased from the smart home to city level, priority for health and AAL decreased in tandem. All smart homes that were evaluated had a focus on health and AAL. At the smart community level, four out of five projects had health as a priority, but only one also focused on AAL (Table 9). Three smart cities with the largest-sized projects prioritized health while two also focused on AAL (Table 9). More broadly, smart communities and cities were observed to have more of a focus on mobility, renewable and sustainable energy, and overall safety. However, it has been noted in some projects, such as the smart city in Amsterdam, that although health was not a part of the initial strategy, as environments evolve and expand their service offerings, healthcare will be added to the agenda (31).

**Table 9:** Results of strategic priority for health and AAL in each level of SLE.

**Type**	**Priority for health**,	**Priority for AAL**,
	N^*^/(%)^**^	N^*^/(%)^**^
Home	5/100%	5/100%
Community	4/80%	1 /20%
City	3/60%	2/40%

* *Number of smart living environments that fit the category/sub-category*.

** *Percentage based on total N (5) for each type of smart living environment reviewed*.

#### Funding for the Canadian Smart Living Environment Landscape Compared to the World

Canada has only recently introduced a framework for smart living environments called The Smart Cities Challenge, which began in 2017 with funding results announced in 2019 (32). The Smart Cities Challenge is a pan-Canadian competition, focused on empowering communities to adopt a smart cities approach to improve people's quality of life through innovation, data, and technology (32).

Over 200 communities applied but only 20 were listed as finalists. Out of twenty, nine communities had a focus on healthy living and recreation, two focused on older adults' health, while one specifically proposed AAL in the City of Cote Saint-Luc. The other seven proposals proposed supporting healthy active lifestyles to reduce non-communicable diseases like diabetes, mental health using health data and digital tools for better decision-making support (Table 10). There was a total of $75 million CAD available and four winning spots. When compared to Europe, the EU has provided multiple frameworks and programs for smart city initiatives dating back to 2014. Under the Horizon 2020 framework, the EU funded 15 smart city projects in 2019, providing a total of ~€83 million in funding, which is ~123 million CAD (33).

**Table 10:** Summary of Canadian smart cities challenge finalists.

**Project**	**Location**	**Funding**	**Focus areas**	**Description**
Biigtigong Nishnaabeg	Ontario	$5 M	• Economic opportunity • Empowerment and inclusion	• Revitalize Indigenous language and culture while preparing their K-12 students for the smart technology future • Utilizing open source software and results-oriented approaches to facilitate effective online learning, effective online acquisition of their endangered Nishnaabe language, and revitalization of their culture—all as an enhancement to their brick-and-mortar K-12 curriculum delivery model
Cree Nation of Eastmain	Quebec	$5 M	• Economic opportunity • Empowerment and inclusion	• Addressing the housing shortage crisis, poor quality design and costly construction of homes in Eastmain by developing affordable Net Zero Energy Housing Program offering culturally appropriate design utilizing smart technologies and innovative building techniques
City of Yellowknife	Northwest Territories	$5 M	• Economic opportunity • Healthy living and recreation	• Incorporating technological innovations into lampposts and creating mesh network allowing them to communicate with one another and a central location • Innovations will include smart motion activated lighting, electric vehicle charging stations, data collection and monitoring, interactive tourism info and Wi-Fi hotspot
Mohawk Council of Akwesasne	Quebec	$5 M	• Healthy living and recreation • Mobility	• Utilizing smart technologies such as electric vehicles, smart greenhouses and integration of mobile/web systems to achieve positive change in lifestyle, education and accessibility to reduce the prevalence of new cases of diabetes
Town of Bridgewater	Nova Scotia	$5 M	• Environmental quality • Empowerment and inclusion	• Implement sophisticated energy monitoring and communications equipment in low-income homes • Develop self-funding energy retrofit financing program • Improve transportation systems • Increase local tech sector training and literacy
The Pas, Opaskwayak Cree Nation, Rural Municipality of Kelsey	Manitoba	$10 M	• Healthy living and recreation • Safety and security	• LED Smart Farm technology will be implemented to support local nutritious food growth and promote food security • Creation of a smart phone distribution system and integration of wearable technology to achieve a reduction in the number of imported vegetables and a reduction in community diabetes rates
City of Cote Saint-Luc	Quebec	$10 M	• Environmental quality • Healthy living and recreation	• Aims to address a rapidly aging population by implementing a connected framework, leveraging smart devices and related technologies that will empower seniors to live more safely and independently in their homes, be better connected to their communities and city services, be more socially engaged • Improving the overall wellbeing and quality of life for older adults and reducing stress on families and caregivers, the healthcare system, and long-term care facilities.
Nunavut Communities	Nunavut	$10 M	• Empowerment and inclusion • Healthy living and recreation	• Development and implementation of decentralized and community-based digital health application intervention called “The Community, Connectivity, and Digital Access for Suicide Prevention” which aims to reduce the risk of suicide • This platform will leverage digital access and connectivity to increase the availability and accessibility of mental health resources and support systems like peer to peer networks, educational initiatives, and creative outlets to all Nunavummiut. This includes an Inuktitut based digital literacy curriculum, improved and innovative network infrastructure, mobile applications, gamified interventions, digital art therapy, and permanent makerspaces available in each community
St. Mary's First Nation and Fredericton	New Brunswick	$10 M	• Empowerment and inclusion	• Recognizing what's important to individuals and connecting them to what matters most • Create a community that is accessible, welcoming, supportive community, starting with youth, newcomers, older adults, and persons with mobility-related disabilities • Empowering residents with personalized digital tools, data & technology that enable them to create an exceptional quality of life
Parkland, Brazeau Lac Ste Anne and Yellowhead Counties	Alberta	$10 M	• Empowerment and inclusion • Economic opportunity	• Aim to transform how rural Canada uses and accesses Information Communications Infrastructure to lever the benefits of connected technologies to improve rural lives, rural economies and rural environments • Greater technology adoption and proper decision support tools will help build more prosperous market and knowledge links with urban Canada and beyond
Greater Victoria	British Columbia	$10 M	• Empowerment and inclusion • Mobility	• Collaboratively create a multimodal transportation network that is convenient, green and affordable increasing mobility wellbeing
City of Guelph and Wellington County	Ontario	$10 M	• Empowerment and Inclusion • Economic opportunity	• Canada's first technology-enabled Circular Food Economy • Reimagining an inclusive food-secure ecosystem that increases access to affordable, nutritious food, where “waste” becomes a resource
City of Saskatoon	Saskatchewan	$10 M	• Empowerment and inclusion • Safety and security	• Use innovative technology to strengthen and connect the supports for youth to grow in a positive learning cycle focused on building purpose, belonging, security and identity and break the cycle of Indigenous youth incarceration
City of Richmond	British Columbia	$10 M	• Mobility • Safety and security	• Develop and implement an integrated platform enabling data driven decision making to improve emergency response rates and reduce recovery time
City of Airdrie and Area	Alberta	$10 M	• Empowerment and inclusion • Healthy living and recreation	• Create an open data platform for use by all by leveraging, connecting existing and adding new infrastructure, platforms and applications to enable informed action to create a healthy community
Waterloo Region	Ontario	$50 M	• Empowerment and inclusion • Healthy living and recreation	• Create framework for data-driven, adaptive and scalable programs that improve early child development, mental health and high school graduation rates • Build Canada's first real-time child and youth wellbeing dashboard that connects data from multiple organizations
Quebec City	Quebec	$50 M	• Environmental quality • Healthy living and recreation	• Utilizing collective intelligence and deployment of digital tools that support decision-making and follow-ups to increase sustainable health and wellbeing
City of Edmonton	Alberta	$50 M	• Empowerment and Inclusion • Healthy living and recreation	• Creating of a Health Data Repository, connecting data from many stakeholders and new technologies to facilitate assessment, analytics and data mining • New municipal health support through digital tool and devices, allowing them to identify and access services, relationships and technologies to improve health and connectedness
City of Surrey and City of Vancouver	British Columbia	$50 M	• Mobility • Safety and security	• Advancing smart mobility infrastructure by implementing Canada's first two collision-free multi-modal transportation corridors, leveraging autonomous vehicles and smart technologies to create safer, healthier and more socially connected communities while reducing emissions, improving transportation efficiency and enhancing livability in the face of rapid growth and traffic congestion
Montreal	Quebec	$50 M	• Mobility • Environmental quality	• Addressing systemic issues of urban life including mobility and access to food • Use technology to implement efficient and sustainable transportation alternatives (car sharing on-demand, autonomous vehicles, bike sharing, etc.) • Innovative transportation alternatives will reinforce the access to local services, most notably to food supply

*Adopted from Infrastructure Canada, Government of Canada (https://www.infrastructure.gc.ca/cities-villes/profiles-profils-eng.html)*.

Canada is currently behind other comparable countries in technology deployment, which is primarily limited to the research and development phase (34). In contrast, the European AAL Programme was introduced by the European Commission to advance innovative research and services for older adults and supports 17 countries with a funding pool of €700 M (35). Canada is now involved in the European AAL Programme, to continue advancing the Canadian AAL landscape.

## Discussion

When developing smart living environments, public consultations, and methodologies to frame user-guided iterations of service offerings are critical. This process can increase user acceptance, trust, and adoption. A growing number of studies have emphasized the importance of participatory and user-centered design since decisions on solutions' design, made independently of users can reduce user acceptance (36–38). Yet, a technocentric approach still seems to persist in AAL developments, resulting in solutions that are high-tech but have low impact for end users (39–41). Participatory design and collaboration among the different stakeholders and end users need to be reinforced in projects and policies when developing smart living environments. This approach will help assure the public that integrative smart technologies in living environments are there to complement and support their daily living and not replace human autonomy or capacity.

Furthermore, there is currently no standardization as it relates to privacy and security policies around the world. In Europe, the cities of Barcelona and Amsterdam both utilize The General Data Protection Regulation (GDPR), which is a regulated by the EU and is centered on data protection and privacy. It is regarded as one of the toughest privacy and security laws in the world that deals with the transfer of personal data outside the EU and European Economic Areas [EAA] (40). In comparison, the cities of Edmonton and Drayton Valley in Canada have adopted the Freedom of Information and Protection of Privacy Act (FOIP Act), which is a legislation enforceable only in the province of Alberta.

Interoperability and accessibility standards pose challenges for the development and implementation of smart living environments. As AAL environments are primarily targeted toward older adults and aging populations with diverse needs, accessibility is an area that requires more dedicated attention and advancement. For interoperability to work well, a wide network of service provider stakeholders are required to collaborate on projects to create a product that brings value to end users. However, due to a lack of technical standards, there are challenges when scaling smart living environments and conducting iterations when adding new features and services. In addition, without interoperable standards, vendor lock-in can cause problems for end users such as the inability to consume products that meet all their needs. Vendor lock-in can also drive-up costs and make services inaccessible for users.

Lastly, it's important to recognize that the work contained in this review is limited to materials published in the English language due to the scope of the research project. As such, it is possible that the results cannot be generalized to materials published in other languages and some studies may have been missed.

## Conclusion

While there have been rapid advancements in technologies to address challenges related to an aging population and support theories active and successful aging, there is a limited amount real-world deployment of AAL technologies, despite the potential benefits they provide to users. There remain wide gaps and challenges when it comes to scaling and integrating AAL into larger environments such as communities and cities. Most communities are more concerned with addressing areas such as mobility and, renewable and sustainable energy. Canada lags behind in AAL deployment compared to other countries around the world like Europe and Asia. As the research and development process of AAL projects often requires a heavy up-front investment and are primarily led by academic institutions, funding may not be sustainable for long periods of time. Providing sustainable and monetary incentives, such as large pools of funding with the focus of AAL can help increase the innovation, development, and implementation.

## Implications

The findings of this study are currently being used in a novel research project that is focused on exploring the continuum between AAL technologies, AAL services, and smart communities. The goal of this project is to develop guidelines for the implementation of smart homes and smart communities that fully leverage the benefits of independent living supported by AAL technology, for use by (i) new AAL technology manufacturers, (ii) developers of new smart communities, and (iii) existing communities seeking to leverage data generated by AAL and IoT sensors into fully integrated community health services. These guidelines will be the product of not only an examination of existing literature, such as what is analyzed in this study, but also focus groups and interviews with relevant actors in the health care sector. An essential component of these guidelines will be the development of a framework for data governance. AAL technologies generate mass amounts of home care-recipient data. Thus, it is important to implement a data governance framework that will ensure the privacy and security of the end-users are prioritized throughout the continuum of care. To develop the framework, it is necessary to identify factors such as independent and dependent variables, relevant actors, collective problems, internal processes, and social norms within the AAL domain. Understanding these factors will in turn unveil data flow requirements, which regulated and unregulated AAL technologies are needed, and how these requirements change as the factors change. With the prioritization of AAL emerging in Canada, such as the newly established Smart Cities Challenge initiative, and involvement in the European AAL Programme, the creation of a data governance framework is critical for the development of smart communities as AAL systems become more complex and integrate more actors. If available, the outcomes of research projects in the European AAL Programme will be considered for the development of guidelines in the subsequent research project.

## Author Contributions

LL, PM, and LF: research concept and methodology. LL, AS, and GB: original draft. PM, LF, and GB: review and editing. PM and LF: supervision. All authors contributed to the article and approved the submitted version.

## Conflict of Interest

The authors declare that the research was conducted in the absence of any commercial or financial relationships that could be construed as a potential conflict of interest.

## Publisher's Note

All claims expressed in this article are solely those of the authors and do not necessarily represent those of their affiliated organizations, or those of the publisher, the editors and the reviewers. Any product that may be evaluated in this article, or claim that may be made by its manufacturer, is not guaranteed or endorsed by the publisher.
